# The Water Supply of Buffalo

**Published:** 1904-09

**Authors:** 


### The Water Supply of Buffalo.

THE report of George W. Fuller, the expert, called to Buffalo to study its water supply has been received and is now before the Common Council. It is the result of personal observation and of the labors of the department of health covering a study of water conditions for the past twelve years and is, therefore, a very complete and careful exposition of our water conditions. It is now the duty of the board of health (Mayor, Health Commissioner and Superintendent of Public Works) to act upon these recommendations and give to the city the practical results of Mr. Fuller's observations.

It is to be regretted that one of the possible sources of water contamination was overlooked—namely, the oft asserted leakage into the new intake tunnel. It seems as if this matter could easily be eliminated, once for all, if the superintendent of public works would give it his special attention.

Some of the conclusions and recommendations of Mr. Fuller deserve careful consideration. The enormous daily consumption of water per capita (324 gallons daily) is commented on and economy urged. But do we consume that amount of water daily? That is a question not at all proven and it is plainly up to the water department to show this pumpage by other methods than by reading off the indicator at the pumping station.

There is either enormous leakage, which can be easily determined, or else we are not using as much water as Mr. Fuller thinks we are. The meterage of water to private families should never be thought of in Buffalo. The subject of filtration is utterly out of the question with a water consumption per capita such as we are supposed to have.

The contamination of the river by South Buffalo sewage through Smoke's Creek and also by the harbor is admitted, as was strongly suspected by water observers ever since the typhoid epidemic at the steel plant colony. The extension of the intake to Horse Shoe reef is urged. It is doubtful if this extension would materially affect the conditions permanently. An extension of the intake toward Windmill Point might be of permanent benefit as far as local conditions are concerned, but it would be an expensive improvement and less efficient than a filtration plant.

What should be done at present, first, is to establish the facts regarding waste water or water consumption and apply the remedy; second, determine as to the leakage of sewage into the intake tunnel and stop it at once; third, make some practical use of the Dodge Street reservoirs, possibly with the idea of sedimentation.

The water question is by no means solved by this report. What has been gained is only a *more emphatic* statement of our water conditions by one more experienced in these matters, but it still remains for us to keep the question alive by study, observation, and constant prodding at those in authority.

As was suggested by a writer before the Academy of Natural Sciences last winter, a commission with power, patterned after the grade crossing commission, appointed by the mayor, holding office indefinitely, could solve this question to better advantage than the board of health as now constituted. The arguments in favor of the commission were given at that time and Mr. Fuller's report could receive at the hands of such a body the necessary consideration commensurate with its importance. The only advice to heed, and that for some time to come, is that given by the health department, "Boil the water for home consumption."

